# Identification and Validation of the N6-Methyladenosine RNA Methylation Regulator YTHDF1 as a Novel Prognostic Marker and Potential Target for Hepatocellular Carcinoma

**DOI:** 10.3389/fmolb.2020.604766

**Published:** 2020-12-10

**Authors:** Saiyan Bian, Wenkai Ni, Mengqi Zhu, Qianqian Song, Jianping Zhang, Runzhou Ni, Wenjie Zheng

**Affiliations:** ^1^Department of Gastroenterology, Affiliated Hospital of Nantong University, Nantong, China; ^2^Research Center of Clinical Medicine, Affiliated Hospital of Nantong University, Nantong, China; ^3^Endoscopy Center and Endoscopy Research Institute, Zhongshan Hospital, Fudan University, Shanghai, China; ^4^Department of Radiology, Wake Forest School of Medicine, One Medical Center Boulevard, Winston-Salem, NC, United States

**Keywords:** m^6^A methylation, regulators, hepatocellular carcinoma, prognosis, YTHDF1, molecular target

## Abstract

**Purpose:** N6-methyladenosine (m^6^A) RNA methylation has been implicated in various malignancies. This study aimed to identify the m^6^A methylation regulator-based prognostic signature for hepatocellular carcinoma (HCC) as well as provide candidate targets for HCC treatment.

**Methods:** The least absolute shrinkage and selection operator (LASSO) analyses were performed to identify a risk signature in The Cancer Genome Atlas (TCGA) datasets. The risk signature was further validated in International Cancer Genome Consortium (ICGC) and Pan-Cancer Analysis of Whole Genomes (PCAWG) datasets. Following transfection of short hairpin RNA (shRNA) targeting YTHDF1, the biological activities of HCC cells were evaluated by Cell Counting Kit-8 (CCK-8), wound-healing, Transwell, flow cytometry, and xenograft tumor assays, respectively. The potential mechanisms mediated by YTHDF1 were predicted by overrepresentation enrichment analysis (ORA)/gene set enrichment analysis (GSEA) and validated by Western blotting.

**Results:** Overexpression of m^6^A RNA methylation regulators was correlated with malignant clinicopathological characteristics of HCC patients. The Cox regression and LASSO analyses identified a risk signature with five m^6^A methylation regulators (KIAA1429, ZC3H13, YTHDF1, YTHDF2, and METTL3). In accordance with HCC cases in TCGA, the prognostic value of risk signature was also determined in ICGC and PCAWG datasets. Following analyzing the expression and clinical implications in TCGA and Gene Expression Omnibus (GEO), YTHDF1 was chosen for further experimental validation. Knockdown of YTHDF1 significantly inhibited the proliferation, migration, and invasion of HCC cells, as well as enhanced the apoptosis *in vitro*. Moreover, silencing YTHDF1 repressed the growth of xenograft tumors *in vivo*. Mechanism investigation indicated that YTHDF1 might promote the aggressive phenotypes by facilitating epithelial–mesenchymal transition (EMT) and activating AKT/glycogen synthase kinase (GSK)-3β/β-catenin signaling.

**Conclusion:** The current study identified a robust risk signature consisting of m^6^A RNA methylation regulators for HCC prognosis. In addition, YTHDF1 was a potential molecular target for HCC treatment.

## Introduction

Hepatocellular carcinoma (HCC) is one of the most common malignancies and ranks the fourth leading cause of deaths worldwide (Bray et al., 2018). The major risk factors of HCC include hepatitis B virus (HBV) and hepatitis C virus (HCV) infection, non-alcoholic steatohepatitis (NASH), alcohol abuse, diabetes mellitus, and aflatoxin exposure (Makarova-Rusher et al., 2016). Exposure to these factors and genetic and epigenetic alterations progressively promote the initiation of HCC (Cancer Genome Atlas Research, 2017). Currently, surgery resection, liver transplantation, and systematic therapy are conventional therapies for HCC. Despite progression in therapeutic strategies for HCC, the overall survival (OS) remains unsatisfactory due to a high rate of postsurgical recurrence and metastasis (Finn et al., 2018). Therefore, it is of great need to elucidate the underlying molecular mechanisms and exploring more novel targets for HCC.

N6-methyladenosine (m^6^A) is the most abundant posttranscriptional modification for eukaryotic mRNA. m^6^A is enriched in the stop codon, 3′ untranslated region (UTR), and long internal exon with average 1–2 m^6^A residues/1,000 nucleotides (Meyer et al., 2012). m^6^A regulates the expression of target genes through the modification of RNA, such as splicing, degradation, exporting, and folding (Wang et al., 2014, 2015). m^6^A can be catalyzed and removed by methyltransferase complexes (MTCs) and demethylases, respectively, which are vividly named as “writers” and “erasers.” Writer proteins include KIAA1429, METTL3, METTL14, RBM15, ZC3H13, and WTAP, in which METTL3 methyltransferase is considered as the key catalytic subunit (Liu et al., 2014). Erasers consist of two demethylases FTO and ALKBH5. There is another type of m^6^A regulators, called “readers,” which consist of HNRNPC and YT521-B homology (YTH) family members (e.g., YTHDF1-3 and YTHDC1/2). These readers could recognize distinct subsets of m^6^A-modified mRNAs specifically and facilitate the regulation of gene expression (Liao et al., 2018). In addition to these classical m^6^A regulators, recent studies have identified some new regulators, such as METTL16 (Warda et al., 2017) and HNRNPA2B1(Alarcon et al., 2015). Interactions among these m^6^A regulators have been implicated in diverse physiological functions and processes, including histogenesis, stem cell self-renewal capacity, and fate determination (Liu et al., 2017). More importantly, increasing studies demonstrate that aberrant m^6^A methylation is correlated with tumorigenesis and progression in multiple cancer types, which functions as either a tumor promoter or a tumor suppressor in distinct states (He et al., 2019).

Some studies indicated that m^6^A regulators were related to poor prognosis of HCC patients and promoted the malignant phenotypes of HCC cells. For example, KIAA1429 was shown to facilitate cell proliferation and invasion of HCC cells through m^6^A modification of ID2 mRNA and GATA3 pre-mRNA (Cheng et al., 2019; Lan et al., 2019). WTAP-mediated m^6^A modification contributed to the aggressiveness of HCC cells *via* posttranscriptional suppression of ETS proto-oncogene 1 (Chen Y. et al., 2019). Interestingly, YTHDF2 was described as an HCC suppressor by repressing cell proliferation *via* m^6^A modification of epidermal growth factor receptor (EGFR). YTHDF2 also inhibited vascular reconstruction and metastasis *via* regulating interleukin 11 and serpin family E member 2 (Hou et al., 2019; Zhong et al., 2019). Moreover, METTL3-mediated m^6^A modification could decrease the expression of suppressor of cytokine signaling 2 in HCC cells, thereby contributing to aggressive phenotype *in vitro* and *in vivo* (Chen et al., 2018). Despite these studies, the clinical significance of these m^6^A regulators in HCC remains unclear and poorly explored. In this study, we aimed to investigate the expression characteristics and clinicopathological value of the m^6^A RNA regulators comprehensively in order to identify robust risk signatures for HCC prognosis and potential targets for HCC treatment.

## Materials and Methods

### Data Acquisition

The RNA-seq transcriptome data of liver hepatocellular carcinoma (LIHC) cohort and corresponding clinical or prognostic information were obtained from TCGA (https://cancergenome.nih.gov/) through the R package “TCGA-Assembler” (Table 1). The genomic alterations of YTHDF1 were identified by cBioPortal (www.cbioportal.org). The YTHDF1 mRNA profiles were also obtained from the International Cancer Genome Consortium (ICGC) and Pan-Cancer Analysis of Whole Genomes (PCAWG) datasets (www.icgc.org) with Gene Expression Omnibus (GEO) datasets including GSE22058, GSE25097, GSE36376, GSE46444, GSE54236, GSE63698, GSE64041, and GSE76427.

**Table 1:** The clinical characteristic information of the HCC patients in TCGA.

**Characteristics**	**Number of cases**	**Percentages (%)**
**Age**		
<65	223	59.63
≥65	150	40.11
Not available	1	0.26
**Gender**		
Male	253	67.65
Female	121	32.35
**Survival status**		
Alive	238	63.64
Dead	130	34.76
Not available	6	1.60
**Stage**		
I	173	46.26
II	87	23.26
III	85	22.73
IV	5	1.34
Not available	24	6.42
**Histological grade**		
G1	55	14.71
G2	178	47.59
G3	124	33.16
G4	12	3.21
Not available	5	1.34
**T classification**		
T1	183	48.93
T2	95	25.40
T3	80	21.39
T4	13	3.48
Not available	3	0.8
**N classification**		
N0	254	67.91
N1	4	1.07
NX	115	30.75
Not available	1	0.27
**M classification**		
M0	268	71.66
M1	4	1.07
MX	102	27.27

*HCC, hepatocellular carcinoma; TCGA, The Cancer Genome Atlas*.

### Selection of N6-Methyladenosine RNA Methylation Regulators

Currently, 13 genes (*KIAA1429, METTL3, METTL14, RBM15, ZC3H13, FTO, ALKBH5, YTHDF1, YTHDF2, YTHDC1, YTHDC2, HNRNPC*, and *WTAP*) are considered as classical m^6^A RNA methylation regulators. To ensure comprehensiveness, we also incorporated three newly acknowledged m^6^A RNA methylation regulator genes (*YTHDF3, METTL16*, and *HNRNPA2B1*). The expression profiles of the above genes were extracted from TCGA LIHC cohort with corresponding clinical information. Heatmap and Vioplot were conducted to visualize the differential expressions of these genes in HCC. The protein–protein interactions (PPIs) among m^6^A RNA methylation regulators were analyzed by STRING database (http://string-db.org). In addition, we performed the Pearson correlation analysis to identify the association among these m^6^A RNA methylation regulators.

### Consensus Clustering Analysis

To further explore m^6^A RNA methylation regulators in the LIHC cohort, we applied consensus clustering analysis to the LIHC cohort based on m^6^A RNA methylation regulators. Two subgroups were identified in the LIHC cohort. In addition, to identify the potential function and involved pathways, we conducted Gene Ontology (GO) and Kyoto Encyclopedia of Genes and Genomes (KEGG) analyses based on the different gene profiles of the two subgroups.

### Prognostic Signature Generation

The correlation of m^6^A RNA methylation regulator genes with OS of HCC patients was evaluated by univariate Cox regression model. A risky gene was characterized by hazard ratios (HRs) > 1, while HRs < 1 were considered a protective one. A five-gene risk signature (KIAA1429, ZC3H13, YTHDF1, YTHDF2, and METTL3) was identified based on the minimum criteria. In addition, risk score was calculated according to the coefficients in the least absolute shrinkage and selection operator (LASSO) algorithm. TCGA LIHC cohort was classified into high- or low-risk group based on the median value of the risk scores.

### Genomic Alteration and Co-expression Gene Identification

The mutation, copy number variation (CNV), and mRNA alterations of YTHDF1 in HCC were analyzed by using the cBioPortal tool (http://cbioportal.org) (Gao et al., 2013). The OncoPrint presented an overview of genetic alterations of YTHDF1 in LIHC samples. Co-expression analysis was determined by using LinkedOmics platform (Vasaikar et al., 2018). The potential function was predicted by overrepresentation enrichment analysis (ORA) with GO_BP/CC/MF, KEGG pathways, Wiki pathway, and Reactome pathway.

### Evaluating the Prognostic Value of the Gene Signature

The distribution of clinicopathological features (age, gender, grade stage, and survival state) was further evaluated in high- and low-risk groups calculated by chi-square test and visualized with heatmaps. Kaplan–Meier analysis with log-rank test was conducted to calculate the difference of OS between patients at high-risk score group and low-risk score group. Receiver operating characteristic (ROC) curve was constructed to evaluate the prognosis value of the signature in predicting the survival of patients. Univariate and multivariate Cox regression analyses were used to evaluate the risk score as an independent prognostic factor of HCC patients.

### Cell Culture and Transfection

Hep3B, HepG2, MHCC97H, MHCC97L, and HCCLM3 were purchased from the Cell Bank of the Chinese Academy of Sciences (Shanghai, China). SMMC7721 and BEL7404 were obtained from American Type Culture Collection (Rockville, MD, USA). Cells were maintained in Dulbecco′s modified Eagle′s medium (GIBCO, USA) supplemented with 10% fetal bovine serum (FBS; GIBCO, USA) and 1% penicillin/streptomycin solution. Plasmids for YTHDF1 knockdown were constructed by Dharmacon (CA, USA). The transfection was performed by Lipo3000 according to the manufacturer′s instruction. The sequences of the short hairpin RNAs (shRNAs) were listed as follows: Kd-YTHDF1-1, GAACAUGCCAGUUUCAAAG; Kd-YTHDF1-2, GGACAGUCAAAUCAGAGUA; Kd-YTHDF1-3, CGACAUCCACCGCUCCAUU; Kd-YTHDF1-4, AAGGAACGGCAGAGUCGAA; NC, UAAGGCUAUGAAGAGAUAC.

### Cell Proliferation Assay

Cell proliferation was evaluated by a Cell Counting Kit-8 (CCK-8; Dojindo Laboratories, Kumamoto, Japan) according to the manufacturer′s introduction. Briefly, HCC cell lines (1,000 cells/well) transfected with control vector or Kd-YTHDF1 plasmids were incubated in 96-well plates for 24 to 96 h. Then, a working solution was administered into the culture medium at 37°C for 2 h. Subsequently, the plates were detected at 450-nm absorbance. Each assay was repeated three times.

### Migration and Invasion Assays

The migration of the HCC cells was evaluated by the wound-healing assay. The HepG2 cells of each group were seeded in the six-well plates. Upon the confluency of 80%, 10-μl tips were used to construct wounds on the surface of each well. Then, the pre-marked places were compared at the indicated time point 0 and 24 h. The distances of migration in three random fields were calculated by ImageJ. The invasion assay was conducted by using the 8-μm Transwell chambers (Corning, Acton, MA, USA) placed in 24-well plates. Here, 200 μl of HepG2 cells were plated in the upper chambers pre-coated with Matrigel (BD, CA, USA), while the complete medium was plated in the lower chamber. Following incubation for 24 h, the chambers were fixed in 4% paraformaldehyde and stained in 0.1% crystal violet solution. Then, the samples of each group were counted under the microscope. Each assay was independently repeated at least three times.

### Flow Cytometry

HCC cells at each group were collected and pre-fixed in 75% cold ethanol and stored at 4°C overnight. After rinsing in phosphate-buffered saline (PBS) three times, the samples were stained in propidium iodide (PI) for 30 min. Subsequently, cell cycle distribution was detected by a flow cytometer (Calibur, Becton Dickinson, CA, USA) and further analyzed by using ModFit Software. The apoptosis detection was conducted by Annexin V-Alexa Fluor 647/PI Apoptosis Detection kit (Fcmacs Biotech Co., Ltd., China) according to the manufacturer′s instruction. The samples were stained in PI and 647 Annexin V for 30 min. Then, the samples were detected by a flow cytometer. Each experiment was independently performed at least three times.

### Xenograft Tumor Assay

Four-week-old BALB/c nude mice were provided by the Animal Center of Nantong University (Nantong, China). HepG2 cells stably transfected with shRNA-YTHDF1 vector or control vector were subcutaneously injected into flanks of nude mice at the density of 5 × 10^6^ cells/100 μl. Post-injection, tumor growth was monitored every 3 days by using calipers. The volume of the xenograft tumor was calculated as 0.5 × length × width^2^. The protocols of this study were approved by the Animal Care and Use Committee of Nantong University.

### Western Blotting

Cells of each group were collected for the extraction of total protein by using radioimmunoprecipitation assay (RIPA) buffer. Then, the samples were separated on a sodium dodecyl sulfate (SDS) gel and transferred onto polyvinylidene difluoride (PVDF) membranes (Bio-Rad, CA, USA). Following blocking in 5% bovine serum albumin (BSA) for 2 h, the membranes were incubated in primary antibodies at 4°C overnight. After rinsing in Tris-buffered saline and Tween 20 (TBST) three times, the samples were further exposed to horseradish peroxidase (HRP)-conjugated secondary antibodies for 2 h at room temperature. The membranes were visualized by using the enhanced chemiluminescence (ECL) kit (Millipore, MA, USA).

### Statistical Analyses

All statistical analyses were calculated by using R software (Version 3.5) and GraphPad Prism 7 (CA, USA). Data are presented as means ± standard deviations. The Student′s *t*-test and chi-square (χ^2^) test were performed to evaluate differences between two groups. A one-way analysis of variance (ANOVA) test was used for multiple group comparisons. The risk score was obtained according to the coefficients in the LASSO algorithm. Kaplan–Meier analysis with a log-rank test was used to analyze the survival difference between the high- and low-risk groups. *P*-value threshold of 0.05 was considered as statistical significance.

## Results

### The Expression Features of N6-Methyladenosine RNA Methylation Regulators

**Figure 1:**
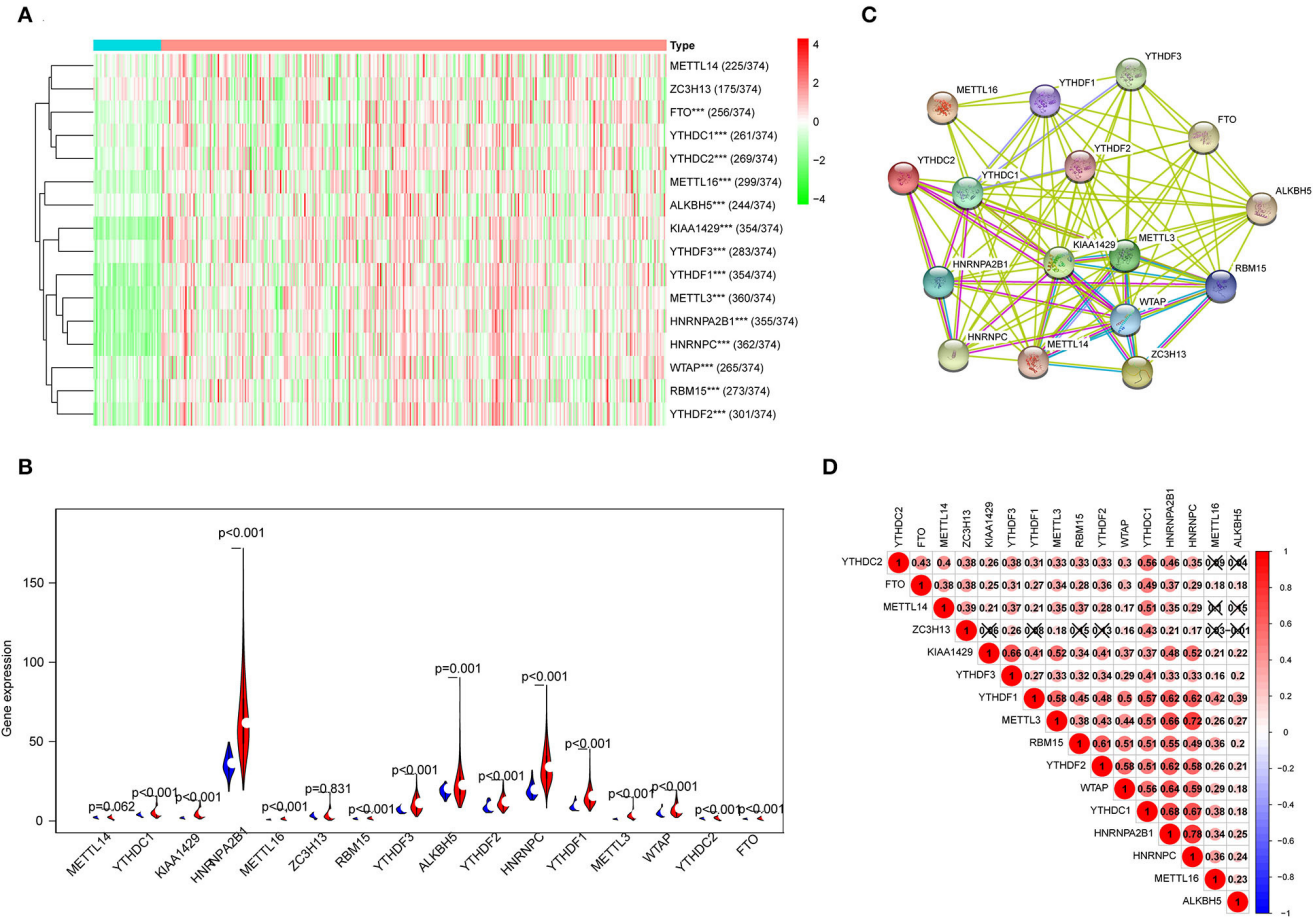
The expression characteristics and correlations of N6-methyladenosine (m^6^A) RNA methylation regulators in hepatocellular carcinoma (HCC). **(A)** Heatmaps presented the overall expression of 16 m^6^A RNA methylation regulators in HCC tissues and normal liver tissues from The Cancer Genome Atlas (TCGA) datasets. **(B)** The differential expression of the m^6^A RNA methylation regulators was visualized by Vioplot (blue means normal liver tissues; red means HCC samples). **(C)** The interaction of the m^6^A RNA methylation regulators was analyzed by STRING. **(D)** Spearman correlation analysis of the 16 regulating genes in the liver hepatocellular carcinoma (LIHC) cohort. ***P < 0.001.

The expression features of 16 m^6^A RNA methylation regulators in HCC tissues and normal liver tissues from TCGA are shown in Figure 1. Compared with normal tissues, 14 m^6^A RNA methylation regulators (YTHDC1, KIAA1429, HNRNPA2B1, METTL16, RBM15, YTHDF3, ALKBH5, YTHDF2, HNRNPC, YTHDF1, METTL3, WTAP, YTHDC2, and FTO) were found overexpressed in HCC (Figures 1A,B). In addition, the interaction network among the 16 m^6^A RNA methylation regulators were predicted by STRING, in which KIAA1429, WTAP, YTHDF2, and METTL3 were considered as hub genes (Figure 1C). Furthermore, the expression of m^6^A RNA methylation regulators were positively correlated based on Pearson correlation. Remarkably, the most relevant among all the m^6^A RNA methylation regulators was observed between the METTL3/HNRNPC (*r* = 0.72) and HNRNPA2B1/HNRNPC (*r* = 0.78) (Figure 1D).

### Correlation of N6-Methyladenosine RNA Methylation Regulators With Clinicopathological Features of Hepatocellular Carcinoma Patients

**Figure 2:**
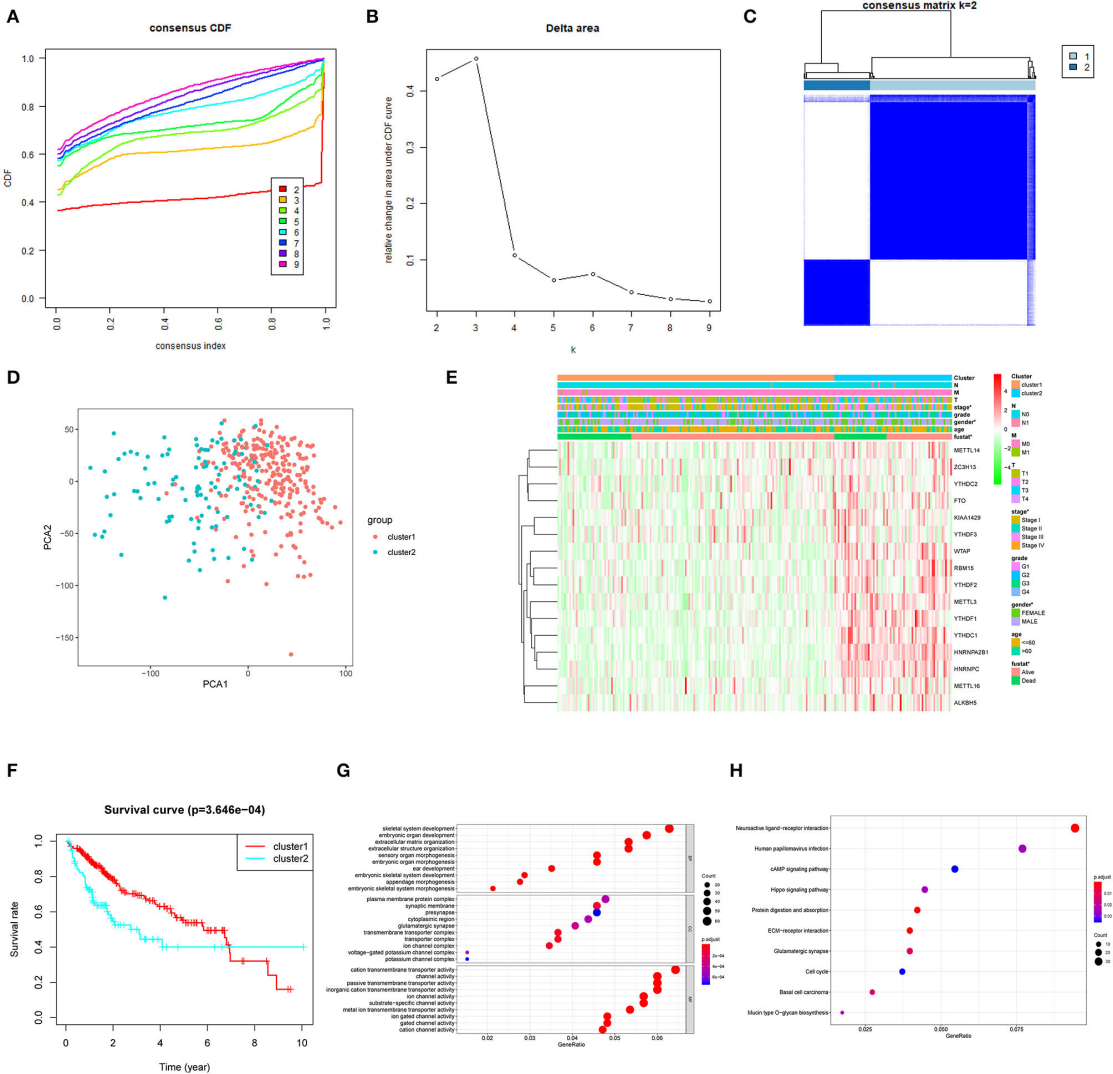
Association between the N6-methyladenosine (m^6^A) RNA methylation regulators and clinicopathological and prognostic features of hepatocellular carcinoma (HCC) patients. **(A)** Consensus clustering model with cumulative distribution function (CDF) for *k* = 2–9. **(B)** Relative change in area under the CDF curve for *k* = 2–9. **(C)** The Cancer Genome Atlas (TCGA) liver hepatocellular carcinoma (LIHC) cohort was classified into two clusters with *k* = 2. **(D)** Principal component analysis of the total RNA expression profile of cluster 1 (red) and the cluster 2 (blue). **(E)** The correlation of the two clusters with clinicopathologic features was visualized by heatmap. **(F)** The overall survival of HCC patients in the two clusters was calculated by Kaplan–Meier curves. **(G)** Gene Ontology (GO) analyses were conducted to predict the potential function of the differentially expressing genes between the two clusters. **(H)** Kyoto Encyclopedia of Genes and Genomes (KEGG) analyses were performed to predict the underlying potential pathways regarding the differentially expressing genes. *P < 0.05.

To develop a prognostic signature based upon m6A RNA methylation regulators, we sought to stratify 374 HCC patient samples by consensus clustering analysis (Figures 2A,B). Based on the cumulative distribution function (CDF) value, *k* = 2 was the optimal cluster number to divide the HCC cohort, namely, cluster 1 and cluster 2 (Figure 2C). Furthermore, principal component analysis (PCA) of total RNA expression profile was performed to evaluate the classification, which showed that cluster 1 and cluster 2 could be well-distinguished (Figure 2D). Next, we evaluated the associations between clusters and clinicopathological features in TCGA. As shown in Figure 2E, the general expression of m^6^A RNA methylation regulators was higher in cluster 2, especially for YTHDC1/2, HNRNPA2B1, METTL16, RBM15, ALKBH5, HNRNPC, YTHDF1-3, METTL3, WTAP, and FTO. In addition, cluster 2 was significantly correlated with gender, advanced stage, and survival state. Moreover, the OS of patients in cluster 2 was significantly lower than that of cluster 1 (Figure 2F). Furthermore, we conducted GO and KEGG analyses based on differentially expressed genes to identify enriched functions and pathways in cluster 2. GO analysis indicated that differentially expressed genes were enriched in various processes, including extracellular matrix (ECM) organization, extracellular structure organization, plasma membrane protein complex, and cation transmembrane transporter activity (Figure 2G). In addition, KEGG analysis indicated that m^6^A RNA methylation regulator-overexpressed cluster 2 was correlated with ECM–receptor interaction, cAMP signaling pathway, Hippo signaling pathway, and cell cycle, which were frequently implicated in the progression of HCC (Massimi et al., 2019; Huang et al., 2020; Wu et al., 2020) (Figure 2H).

### Prognostic Significance of the N6-Methyladenosine RNA Methylation Regulator-Based Signature

**Figure 3:**
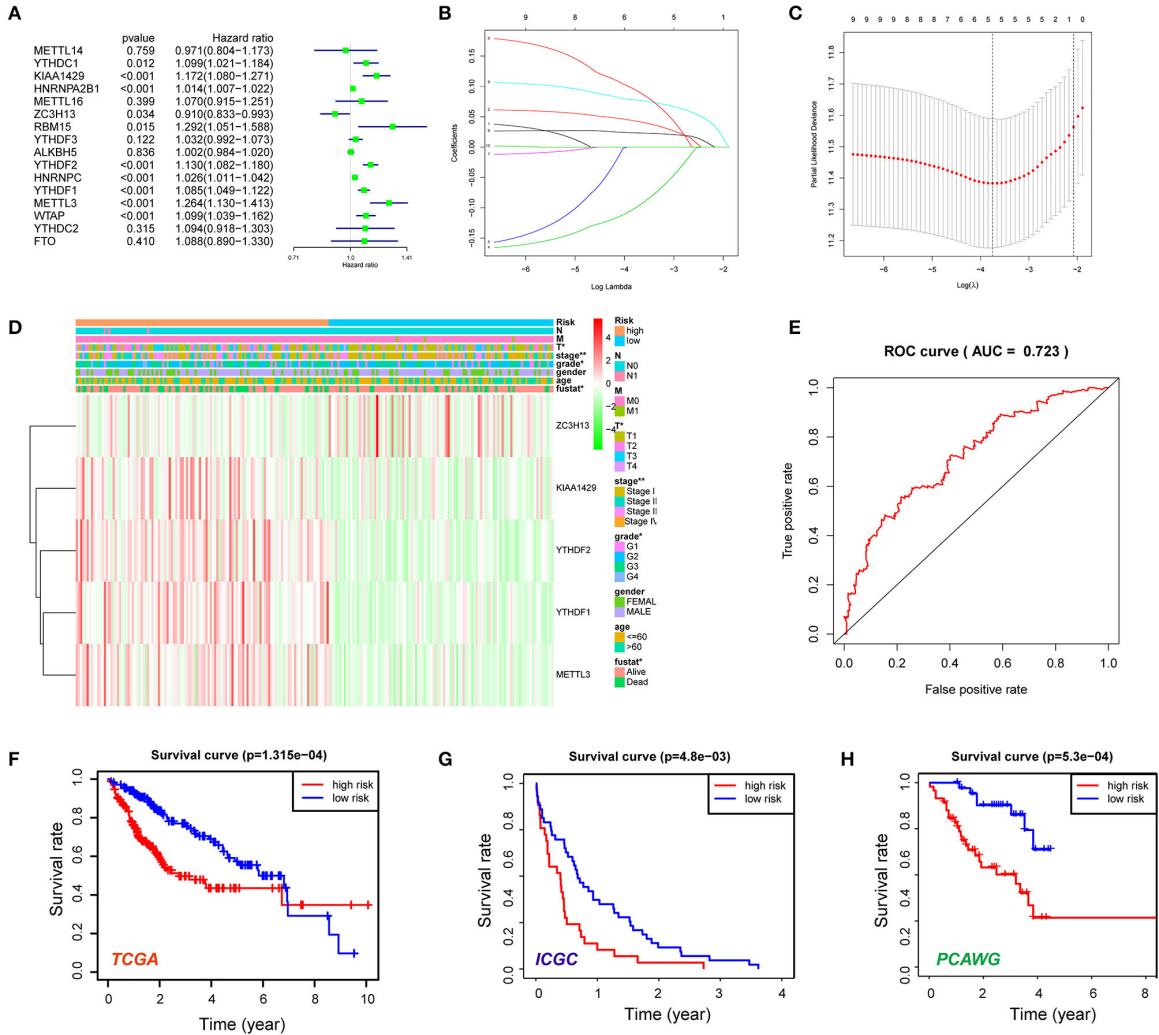
Construction of the five N6-methyladenosine (m^6^A) RNA methylation regulator-based risk signature. **(A)** Univariate Cox regression was performed to screen the signature in 16 m^6^A RNA methylation regulators. **(B,C)** The coefficients of the five-gene signature (KIAA1429, ZC3H13, YTHDF1, YTHDF2, and METTL3) calculated by multivariate Cox regression with least absolute shrinkage and selection operator (LASSO). **(D)** The expression features of the five m^6^A RNA methylation regulators and the distribution of clinicopathological features were compared between the low- and high-risk groups of The Cancer Genome Atlas (TCGA) liver hepatocellular carcinoma (LIHC) datasets. **(E)** Receiver operating characteristic (ROC) curves were calculated to evaluate the predictive efficiency of the five-gene risk signature. **(F)** The Kaplan–Meier curves of HCC patients at the high-risk group and low-risk group in TCGA cohort. **(G)** The Kaplan–Meier curves of HCC patients at the high-risk group and low-risk group in the International Cancer Genome Consortium (ICGC) cohort. **(H)** The Kaplan–Meier curves of HCC patients at the high-risk group and low-risk group in the Pan-Cancer Analysis of Whole Genomes (PCAWG) cohort. *P < 0.05, **P < 0.01.

Then, the prognostic significance of m^6^A RNA methylation regulators was evaluated for HCC patients. The univariate Cox regression and Kaplan–Meier analyses indicated that nine of the regulators were associated with poor survival of the HCC cases (Figure 3A; Supplementary Figure 1), including YTHDC1, KIAA1429, HNRNPA2B1, RBM15, YTHDF2, YTHDF1, HNRNPC, METTL3, and WTAP. In contrast, ZC3H13 was considered as a protective factor for HCC patients. Next, the LASSO algorithm, a generalized linear model, was performed to establish the prognostic signature. A coefficient profile plot was generated after the log2 transformation of the lambda (λ) value, which was determined by the smallest likelihood deviance (Figure 3B). Five m^6^A RNA methylation regulators (KIAA1429, ZC3H13, YTHDF1, YTHDF2, and METTL3) and corresponding coefficients were identified with minimum 10-fold cross-validated mean square error in TCGA cohort (Figure 3C). The risk score for each patient = ∑gene expression * coefficient (glmnet R package). Based on the median of the risk score, we stratified the HCC cohort into high-risk group and low-risk group. High-risk score group was positively correlated with aggressive pathological features like T status, tumor stage, and histological grade (Figure 3D). As shown in Figure 3E, the signature′s risk score could robustly predict survival rates for HCC patients [area under the curve (AUC) = 0.723]. Next, Kaplan–Meier analysis demonstrated that patients in the high-risk group had significantly shorter OS than low-risk cases (*P* < 0.001; Figure 3F). Furthermore, we evaluated the five-gene signature in stratification analyses (Supplementary Figures 2A,B). The high-risk score could predict poor prognosis for HCC patients at early tumor stages (*P* = 0.0018) and histological grades (*P* < 0.001). Although the difference was not statistically significant, the OS of high-risk cases at advanced tumor stages or histological grades was obviously lower than that of the low-risk group. We further conducted the multivariate Cox regression analysis and identified the risk signature as an independent prognostic factor. Consistently, the univariate and multivariate Cox regression analyses demonstrated that the signature-based risk score was an independent factor (*P* < 0.001, HR = 1.166, 95% CI = 1.099–1.236) for predicting the OS of HCC patients (Supplementary Figures 3A,B). In addition, we also evaluated the risk signature in ICGC and PCAWG datasets. In accordance, high-risk score indicated the poor survival of HCC patients in both of the two datasets (Figures 3G,H). The results above indicated that the m^6^A RNA methylation regulators were involved in HCC progression and serve as a potential biomarker for prognosis.

### The Genomic Alteration and Clinical Implication of YTHDF1 in Hepatocellular Carcinoma

**Figure 4:**
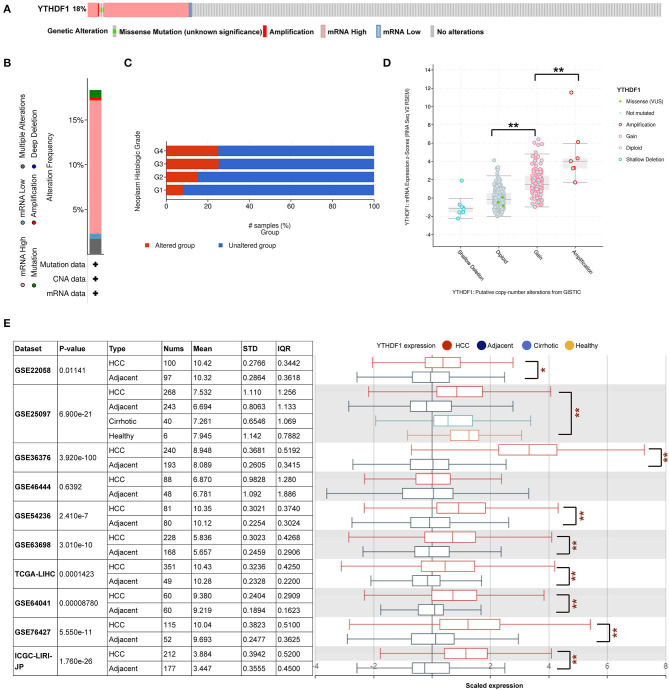
YTHDF1 genomic alterations in hepatocellular carcinoma (HCC). **(A,B)** OncoPrint of YTHDF1 alterations in liver hepatocellular carcinoma (LIHC) cohort identified by cBioPortal. **(C)** YTHDF1 mRNA expression in different YTHDF1 copy number variation (CNV) groups. **(D)** Distribution of YTHDF1 CNV frequency in LIHC cases at different grades. **(E)** YTHDF1 expression in 10 Gene Expression Omnibus (GEO) datasets. *P < 0.05, **P < 0.01.

Given that the m^6^A regulator-based signature was correlated with tumor stage and histological grade, we further evaluated the expression of five regulators in different stages or grades of HCC (Supplementary Figures 4A,B). From early stages (grades) to advanced stages (grades), the expression level of YTHDF1 was remarkably elevated. Thus, we subsequently focused on the m^6^A reader YTHDF1. Initially, the types and frequency of YTHDF1 alterations of YTHDF1 were determined by cBioPortal. According to the OncoPrint (Figures 4A,B), YTHDF1 was altered in 65 of 360 (18.06%) LIHC patients, including mRNA upregulation in 53 cases (14.72%), amplification in one case (0.28%), mutation in three cases (0.83%), and multiple alterations in six cases (1.67%). In addition, the YTHDF1 alteration was enhanced in advanced grades of HCC patients (Figure 4C). Compared with the diploid cases, gain or amplification cases had higher YTHDF1 expression levels (*P* < 0.01; Figure 4D). Next, we conducted a meta-analysis of YTHDF1 mRNA expression in ICGC and GEO datasets (Figure 4E). In most datasets (9/10), HCC tissues presented significantly higher expression of YTHDF1 than that of normal liver tissues. Then, we further evaluated the diagnostic value based on the expression features of YTHDF1 by using ROC curves. As shown in Supplementary Figures 5A–E, YTHDF1, with distinct expression level in contrast to normal tissues, showed a potential diagnostic value in the whole cohort or cases at all different stages. Then, the clinical implications of YTHDF1 was subsequently analyzed in TCGA datasets. Overexpression of YTHDF1 was correlated with tumor volume, distant metastasis, histological grade, and neoplasm stage (Table 2). Furthermore, the univariate analysis and multivariate analysis suggested YTHDF1 as an independent prognostic marker for OS and recurrence-free survival of HCC patients in TCGA (Tables 3, 4).

**Table 2:** Correlation of YTHDF1 expression with clinical features of HCC patients in TCGA.

**Clinical characteristics**	**Total (N)**	**Odds ratio in YTHDF1**	***P*-value**
Age (≥65 vs. <65)	370	1.07 (0.71,1.62)	0.75
Gender (female vs. male)	371	0.76 (0.49,1.17)	0.209
Stage (I/II vs. III/IV)	347	3.27 (1.96,5.47)	**<0.0001**
Histological grade	366	2.49 (1.61,3.87)	**<0.0001**
(G1/G2 vs. G3/G4)			
T (T1/T2 vs. T3/T4)	368	3.2 (1.93,5.32)	**<0.0001**
N (N0 vs. N1)	256	2.68 (0.28,26.15)	0.364
M (M0 vs. M1)	270	68825722.07 (0, Inf)	**0.021**

*Bold, P < 0.05. HCC, hepatocellular carcinoma; TCGA, The Cancer Genome Atlas*.

**Table 3:** Univariate analysis and multivariate analysis of overall survival in HCC patients from TCGA.

**Parameters**	**Univariate analysis**					**Multivariate analysis**		
	**HR**	**CI lower**	**CI upper**	***P***	**HR**	**CI lower**	**CI upper**	***P***
Age	1	1	1	0.079	1.01	0.993	1.02	0.325
Gender	0.82	0.57	1.2	0.262	0.958	0.666	1.38	0.819
Histological grade	1.1	0.85	1.3	0.651	1.15	0.911	1.46	0.238
M	1.3	1.1	1.5	**0.0092**	1.29	1.01	1.66	**0.0416**
N	1.2	1	1.5	**0.0378**	1.1	0.851	1.43	0.455
T	1.2	1.2	1.3	**<0.0001**	1.21	1.1	1.33	**<0.0001**
Pathologic stage	1.2	1.1	1.3	**0.00012**	1.04	0.919	1.17	0.559
YTHDF1	2.7	1.5	4.6	**0.000462**	2.2	1.2	4.03	**0.011**

*Bold, P < 0.05. CI, confidence interval; HCC, hepatocellular carcinoma; HR, hazard ratio; TCGA, The Cancer Genome Atlas*.

**Table 4:** Univariate analysis and multivariate analysis of recurrence-free survival in HCC patients from TCGA.

**Parameters**	**Univariate analysis**					**Multivariate analysis**		
	**HR**	**CI lower**	**CI upper**	***P***	**HR**	**CIlower**	**CI upper**	***P***
Age	1	0.99	1	0.849	0.996	0.983	1.01	0.608
Gender	0.98	0.69	1.4	0.919	1.17	0.811	1.7	0.395
Histological grade	0.98	0.8	1.2	0.873	0.982	0.791	1.22	0.87
M	0.96	0.79	1.2	0.694	0.92	0.704	1.2	0.543
N	1	0.87	1.3	0.656	1.17	0.913	1.51	0.212
T	1.3	1.2	1.4	**<0.0001**	1.23	1.1	1.38	**0.000308**
Pathologic stage	1.3	1.2	1.5	**<0.0001**	1.05	0.888	1.24	0.565
YTHDF1	2.5	1.5	4.3	**0.000581**	2.07	1.18	3.62	**0.0111**

*Bold, P < 0.05. CI, confidence interval; HCC, hepatocellular carcinoma; HR, hazard ratio; TCGA, The Cancer Genome Atlas*.

### The Contribution of YTHDF1 to the Aggressive Behavior of Hepatocellular Carcinoma Cells

**Figure 5:**
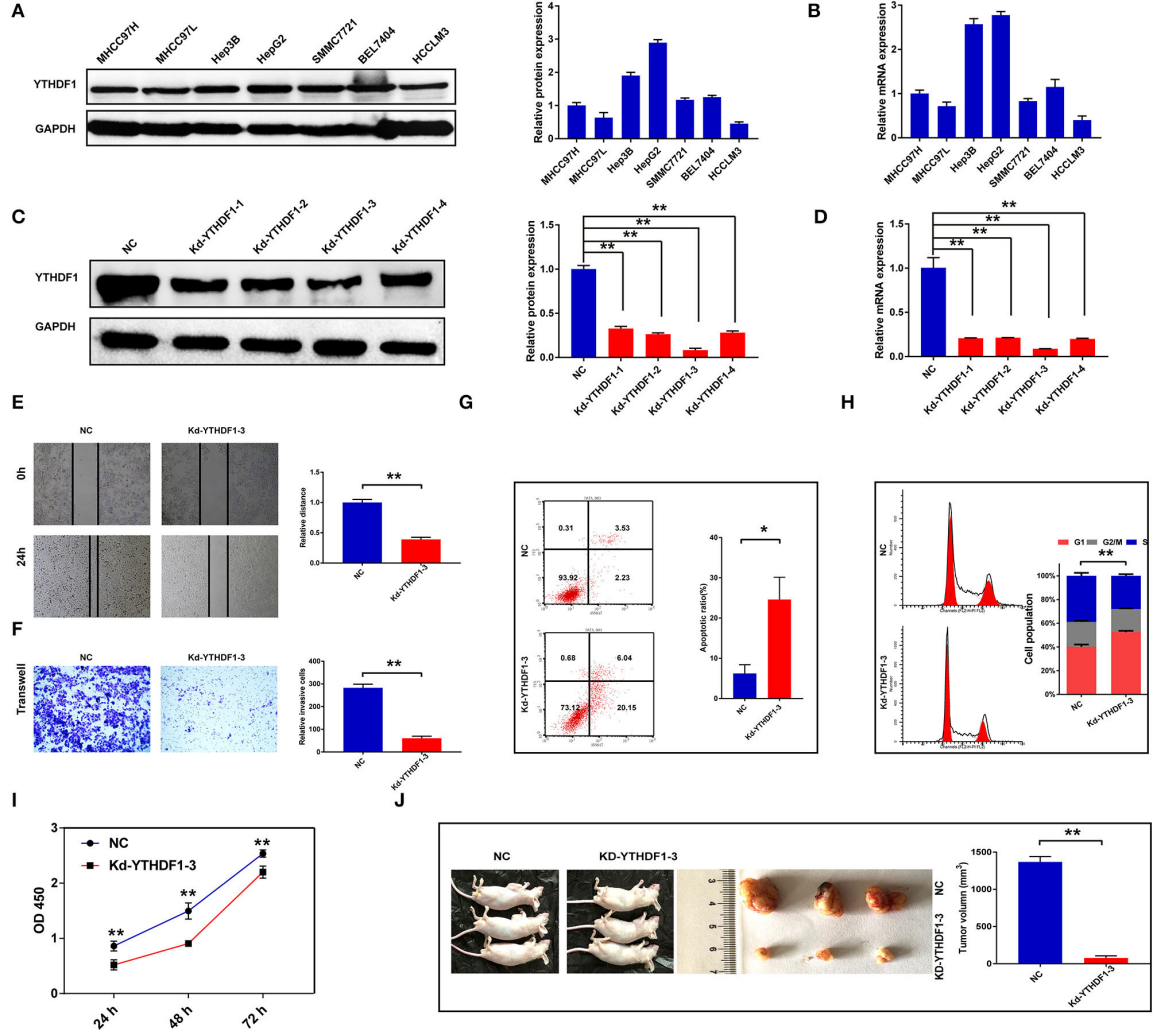
Silencing YTHDF1 inhibited the aggressive behaviors of hepatocellular carcinoma (HCC) cells. **(A)** The expression of YTHDF1 in HCC cell lines was detected by Western blotting. **(B)** The mRNA level of YTHDF1 in HCC cell lines was detected by qRT-PCR. **(C)** Short hairpin RNAs (shRNAs) were transfected into HepG2 cells to block the YTHDF1 expression. The expression of YTHDF1 in each group was detected by Western blotting. **(D)** The mRNA expression of YTHDF1 in each group was detected by qRT-PCR. **(E,F)** Migration and invasion of HepG2 cells were detected by wound-healing assay and Transwell assay. **(G)** The apoptosis of HepG2 cells with YTHDF1 silencing. **(H)** Cell cycle of HepG2 cells was evaluated by flow cytometry. **(I)** The proliferation of cells was detected by Cell Counting Kit-8 (CCK-8). **(J)** The effects of YTHDF1 on tumor growth were evaluated by xenograft tumor in nude mice. *P < 0.05, **P < 0.01.

To further discover the roles of YTHDF1 in HCC, the current study conducted the functional assays *in vitro* and *in vivo*. First, we detected the protein and mRNA expressions of YTHDF1 in seven HCC cell lines, in which HepG2 had the highest expression level (Figures 5A,B). Then, four shRNAs were transfected into HepG2 cells to knock down YTHDF1, and Kd-YTHDF1-3 presented the best inhibitory efficiency (Figures 5C,D). Following knockdown of YTHDF1, the migration (*P* < 0.001) and invasion (*P* < 0.001) were significantly inhibited (Figures 5E,F), while the apoptosis ratio of HepG2 cells was dramatically increased (*P* = 0.0132; Figure 5G). As presented in Figure 5H, the flow cytometry showed that depletion of YTHDF1 increased the proportion of cells in G0/G1. Consistently, the proliferation of HCC cells was significantly repressed in the Kd-YTHDF1-3 group (Figure 5I). Furthermore, knockdown of YTHDF1 obviously decreased the volume of xenograft tumors (*P* < 0.001; Figure 5J). These pieces of evidence suggested the correlation of YTHDF1 with aggressive phenotypes of HCC cells.

### YTHDF1 Regulated Epithelial–Mesenchymal Transition and AKT/Glycogen Synthase Kinase-3β/β-Catenin Signaling of Hepatocellular Carcinoma Cells

**Figure 6:**
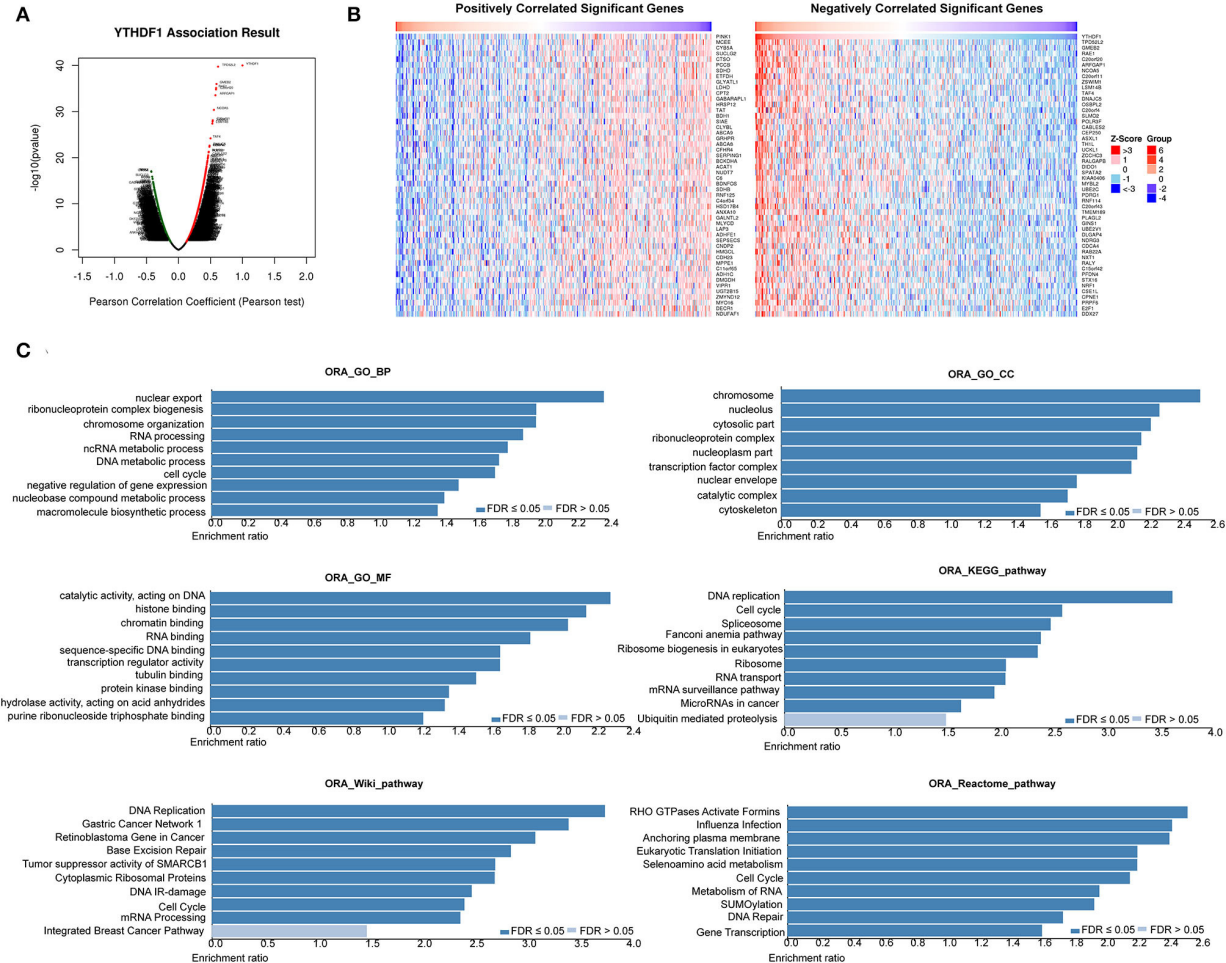
YTHDF1 co-expression genes in hepatocellular carcinoma (HCC). **(A)** The global YTHDF1 significantly correlated genes in the liver hepatocellular carcinoma (LIHC) cohort were identified by LinkedOmics. **(B)** Heatmaps showing top 50 genes positively and negatively correlated with YTHDF1 in LIHC. Red dot, positively correlated gene; blue dot, negatively correlated genes. **(C)** Overrepresentation enrichment analysis of the significantly correlated genes in the LIHC cohort.

**Figure 7:**
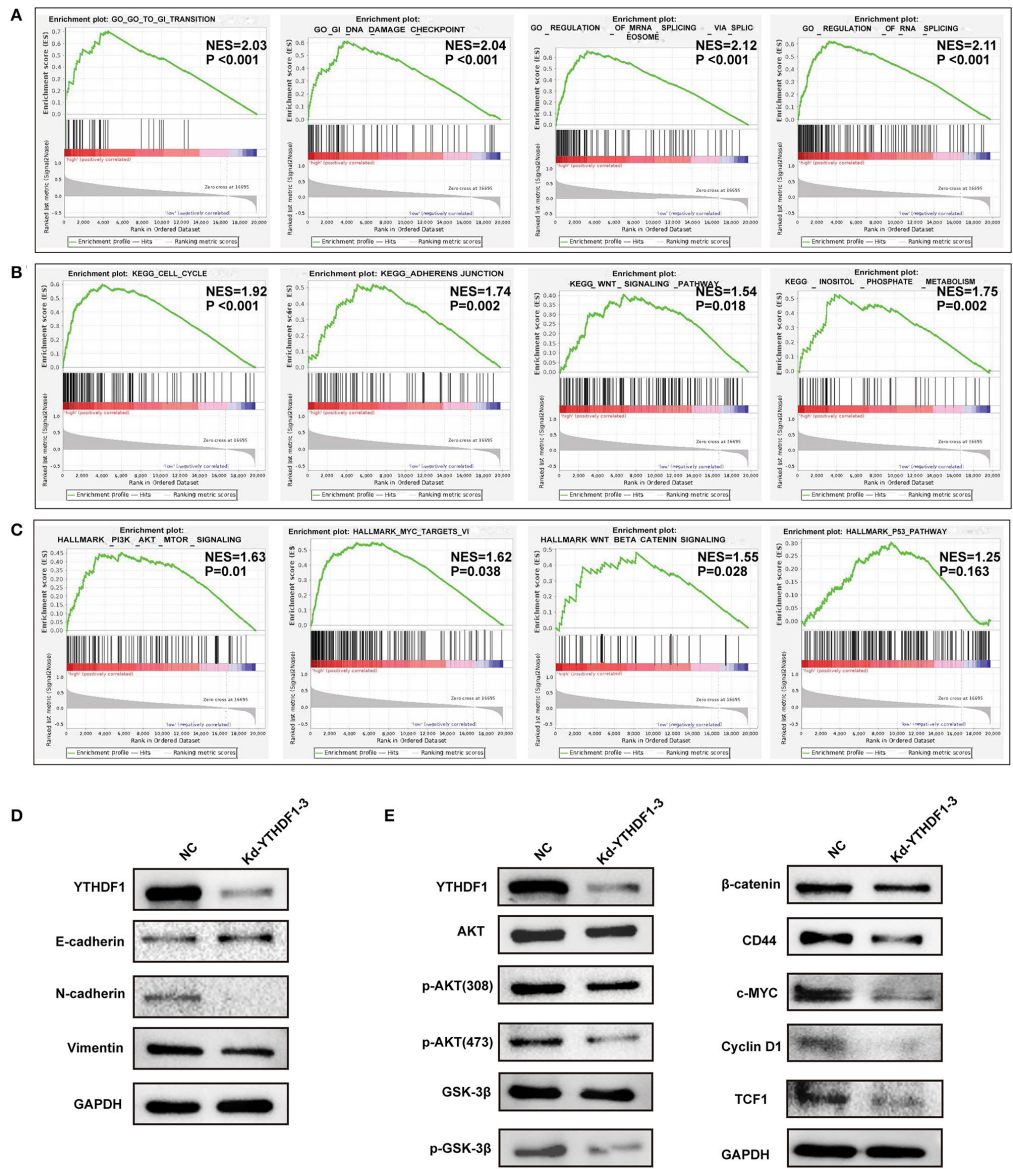
YTHDF1 promoted epithelial–mesenchymal transition (EMT) and AKT/glycogen synthase kinase (GSK)-3β/β-catenin signaling of hepatocellular carcinoma (HCC) cells. **(A–C)** Gene set enrichment analysis (GSEA) was conducted to predict the potential functions and pathways regulated by YTHDF1 based on The Cancer Genome Atlas (TCGA) datasets. **(D)** The EMT markers were detected by Western blotting. **(E)** The markers of AKT/GSK-3β and Wnt/ β-catenin were detected by Western blotting.

Based on the observations above, we further discovered the underlying mechanisms regulated by YTHDF1. In general, co-occurrence genes shared similar functions and mechanisms. Thus, we examined the co-occurrence profiles with YTHDF1 in HCC by LinkFinder. A total of 5,150 in 19,922 genes were defined as positively or negatively correlated significant genes with YTHDF1 (Figures 6A,B). ORA indicated that the co-occurrence genes were implicated in the RNA process, cell cycle, RNA binding, transcription regulator activity, DNA replication, and SUMOylation (Figure 6C). Next, GSEA was conducted to predict the potential functions and pathways induced by YTHDF1. The GO analysis suggested the association between YTHDF1 and G0/G1 transition, G1 damage checkpoint, mRNA splicing *via* spliceosome, and RNA splicing (Figure 7A). In addition, the KEGG analysis indicated that YTHDF1 was correlated with cell cycle, adherens junction, Wnt signaling, and phosphate metabolism (Figure 7B). As shown in Figure 7C, Hallmark analysis showed that YTHDF1 might be implicated in phosphoinositide 3-kinase (PI3K)/AKT signaling, MYC targets, Wnt/β-catenin signaling, and P53 pathway. Then, Western blotting was performed to verify the prediction above. Given the effects on invasion features and bioinformatic prediction, we initially detected epithelial–mesenchymal transition (EMT) markers. As shown in Figure 7D, knockdown of YTHDF1 significantly decreased the expression of N-cadherin and vimentin with the upregulation of E-cadherin, suggesting the positive effects of YTHDF1 on EMT process of HepG2 cells. Subsequently, we detected the pathways predicted by GSEA (Figure 7E). Following silencing YTHDF1, the expression of P-AKT(S308), P-AKT(S473), P-GSK-3β, β-catenin, c-MYC, TCF-1, cyclin D1, and CD44 was significantly downregulated, while the expression of total AKT and GSK-3β had no obvious changes. It suggested that YTHDF1 might enhance the aggressive behaviors of HCC cells through promoting the EMT process and activating AKT/GSK-3β/β-catenin signaling.

## Discussion

It is well-elucidated that the genetic and epigenetic alterations induced by the m^6^A RNA methylation regulators modulate the related phenotypes. Aberrantly expressed m^6^A RNA methylation regulators have been correlated with various malignant behaviors in multiple cancer types. For HCC, previous studies indicated that some m^6^A RNA methylation regulators like KIAA1429, WTAP, and FTO were overexpressed in tissues and cell lines. In this study, we examined 16 widely reported m^6^A RNA methylation regulators in TCGA LIHC datasets. Consistently, most m^6^A regulators were overexpressed in HCC tissues compared with normal liver tissues. Based on the expression of m^6^A regulators, we further divided the HCC cohort into two clusters by consensus clustering. Cluster 2, with high expression levels of m^6^A RNA methylation regulators, showed significantly lower survival and higher tumor grades in contrast to those of cluster 1. It indicated that the expression of m^6^A RNA methylation regulators might be associated with poor prognosis of HCC. According to the univariate Cox regression analysis, 10 of the 16 m^6^A regulator genes were considered potential prognostic factors of HCC. Furthermore, differentially expressed genes between the two clusters were found enriched in well-known tumor-related pathways, including cAMP signaling pathway, Hippo signaling pathway, cell cycle, AMP-activated protein kinase (AMPK) signaling pathway, and PI3K–AKT signaling pathway. It further suggested the underlying correlations of m^6^A methylation regulators with initiation and progression of HCC.

Furthermore, by using LASSO algorithm, we constructed a risk signature with five m^6^A RNA methylation regulators, including KIAA1429, ZC3H13, YTHDF1, YTHDF2, and METTL3. The risk score of this signature was correlated with aggressive clinicopathological features, which could also act as an independent prognosis factor for the survival of patients. In addition, the risk score derived from five m^6^A RNA methylation regulators showed potential prognostic value in patients at different tumor stages. One recent study reported a signature consisting of METTL14 and METTL3 as an independent prognosis factor in clear cell renal cell carcinoma (Wang et al., 2020). Similar results about the clinical value of m^6^A regulators have also been found in bladder cancer, head and neck squamous cell carcinoma, and gastric cancer (Chen M. et al., 2019; Su et al., 2019; Zhao and Cui, 2019). However, different from previous studies, this study, for the first time, validated the prognostic value of the risk signature in two additional datasets, the ICGC and PCAWG datasets. As expected, the m^6^A regulator-based risk signature could predict poor survival of HCC cases in both of the two datasets. Given the current data, further efforts should be put to evaluate m^6^A methylation regulators as robust predictors in more HCC cohorts at multicenter level.

In addition to the prognostic value of the risk signature, the m^6^A RNA methylation regulators might also be associated with tumor progression. Of them, the expressions of YTHDF1 and YTHDF2 are shown to be dramatically elevated in HCC cases from early to advanced stages. As is known, the YTH domain family members can recognize and directly bind m^6^A methylation on RNA. Though YTHDF1 and YTHDF2 share the same m^6^A site-related YTH domain, other domains may bring them distinct functions. In the cytoplasm, YTHDF1 facilitates ribosome loading and promotes target translation, while YTHDF2 localizes its targets to process bodies and induce further degradation. YTHDF2 was recently recognized as a tumor suppressor, in which proliferation and angiogenesis were impaired by YTHDF2 overexpression (Hou et al., 2019; Zhong et al., 2019). In contrast, YTHDF1 promoted malignant behaviors by regulating the translation of tumor-related genes (Liu et al., 2020). A recent study indicated that the aberrant expression of YTHDF1 was associated with poor survival of HCC patients (Zhao et al., 2018). According to the bioinformatic analysis, overexpression of YTHDF1 was observed in the ICGC and multiple GEO datasets. Thus, we further investigated the expression features, potential roles, and mechanisms of YTHDF1 in HCC. As shown in functional assays, silencing YTHDF1 significantly inhibited the malignant behaviors of HCC cells, including proliferation, migration, invasion, and growth of xenograft tumors. Then, the current study tried to investigate potential mechanisms in combination of the bioinformatic prediction and molecular validation. In consistence with the phenotype changes induced by YTHDF1 silencing, GSEA indicated that YTHDF1 might be implicated in cell cycle, G0/G1 transition, and adherens junction. Further molecular assays demonstrated that YTHDF1 could promote EMT of HCC cells. In addition, knockdown of YTHDF1 significantly downregulated the phosphorylation level of AKT and GSK-3β and expression of β-catenin and its downstream markers like CD44, c-MYC, and TCF-1, suggesting that YTHDF1 might enhance aggressive phenotypes by activating AKT/GSK-3β/β-catenin signaling. Interestingly, YTHDF1 was recently reported to facilitate cancer stem cell properties (Bai et al., 2019). β-Catenin and its downstream CD44 were known as canonical markers of liver cancer stem cells. It was speculated that YTHDF1 might be involved in the regulation of cancer stem cell properties, thereby facilitating HCC progression. Though existing pieces of evidence have shed light on the carcinogenic effects of m^6^A RNA methylation regulator YTHDF1, more investigations should be conducted to further reveal its underlying mechanisms as well as the clinical significance in HCC.

## Conclusion

In conclusion, this study established a risk signature formed by five m^6^A RNA methylation regulators for HCC prognosis based on TCGA datasets, which was further validated in ICGC and PCAWG datasets. Furthermore, YTHDF1 was further identified as an oncogenic gene for HCC by facilitating AKT/GSK-3β/β-catenin signaling. Our study provided evidence for future exploration of the prognostic and targeted value of m^6^A methylation in HCC.

## Data Availability Statement

The datasets presented in this study can be found in online repositories. The names of the repository/repositories and accession number(s) can be found in the article/Supplementary Material.

## Ethics Statement

The animal study was reviewed and approved by The Animal Care and Use Committee of Nantong University. Written informed consent was obtained from the owners for the participation of their animals in this study.

## Author Contributions

WZ and RN conceived and designed the study. QS, WN, and JZ analyzed the data. SB and MZ drafted the paper. WZ revised the manuscript. All authors read and approved the final manuscript.

## Conflict of Interest

The authors declare that the research was conducted in the absence of any commercial or financial relationships that could be construed as a potential conflict of interest.
