# Supplementary material for: Identification and Validation of the N6-Methyladenosine RNA Methylation Regulator YTHDF1 as a Novel Prognostic Marker and Potential Target for Hepatocellular Carcinoma
Source: Front Mol Biosci. 2020 Dec 10;7:604766. doi: 10.3389/fmolb.2020.604766 (PMC7758441; doi:10.3389/fmolb.2020.604766)
Supplement: Supplementary file 1 [file Image_1.PDF]

Supplementary Material

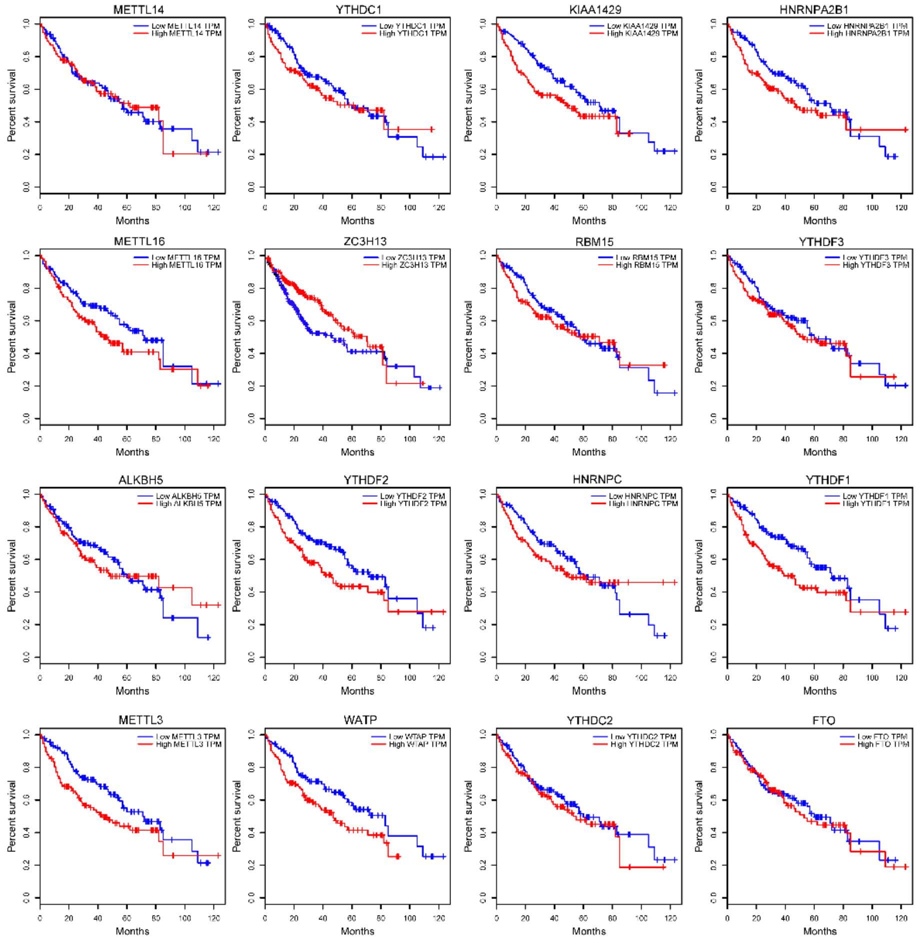

Figure S1. The Kaplan-Meier curves of the 16 m6A regulators in TCGA LIHC cohort. The cohort was stratified into high expression group and low expression group based on the median value of each gene.

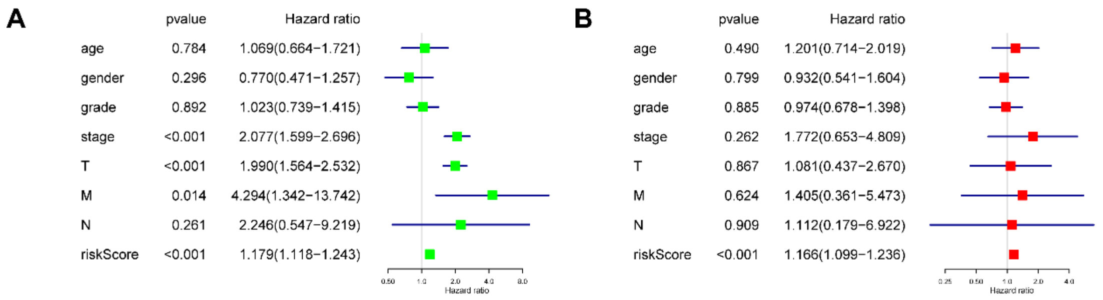

Figure S2. The stratification analysis of the risk signature. (A) The differences of overall survival between the high-risk group and low-risk group in cases at early or advanced tumor stages. **(B)** The Kaplan-Meier curves of the high-risk group and low-risk group in cases at early or advanced tumor grades.

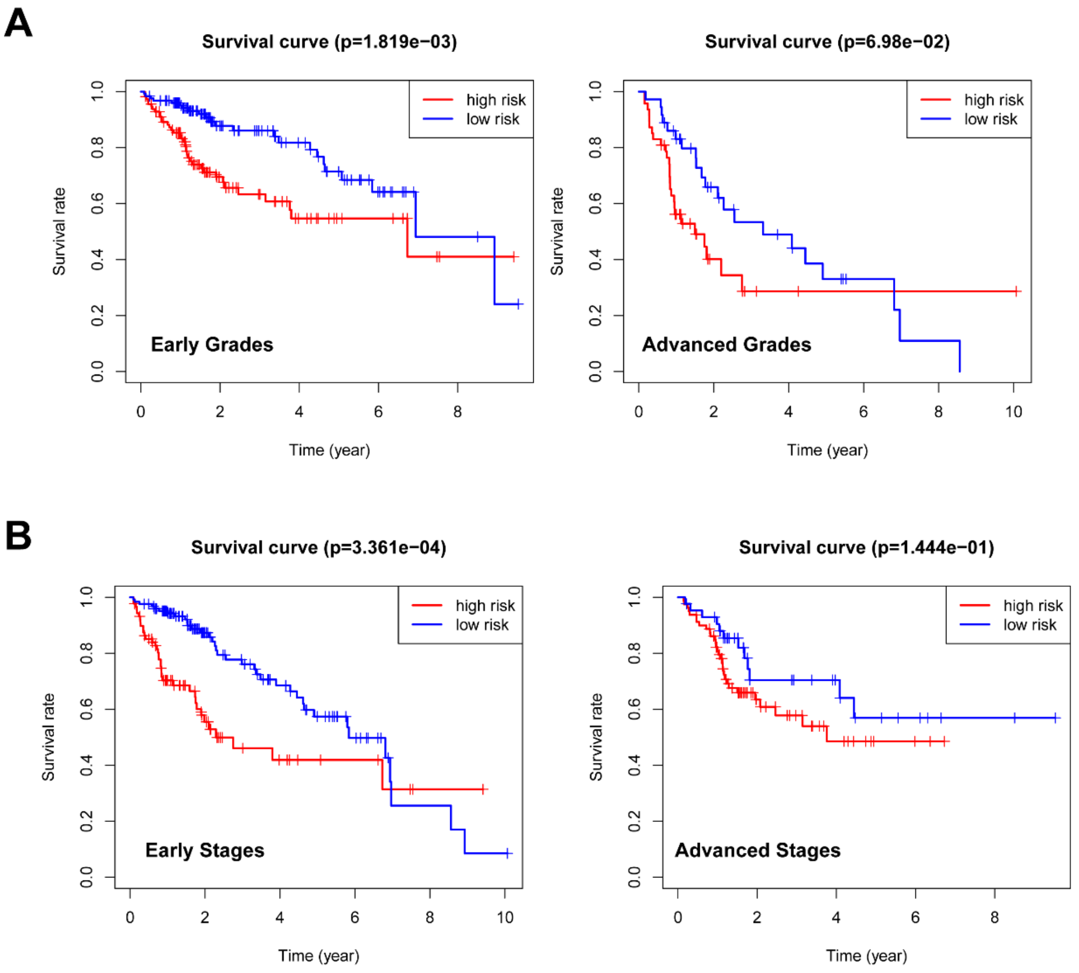

Figure S3. The Univariate and multivariate Cox regression of the risk signature. (A) Univariate Cox regression analyses identified the association between clinicopathological factors (including the risk score) and overall survival of patients in the TCGA datasets. **(B)** Multivariate Cox regression analyses identified the independent factor overall survival of patients in the TCGA datasets. CI, confidence intervals. HR, hazard ratios.

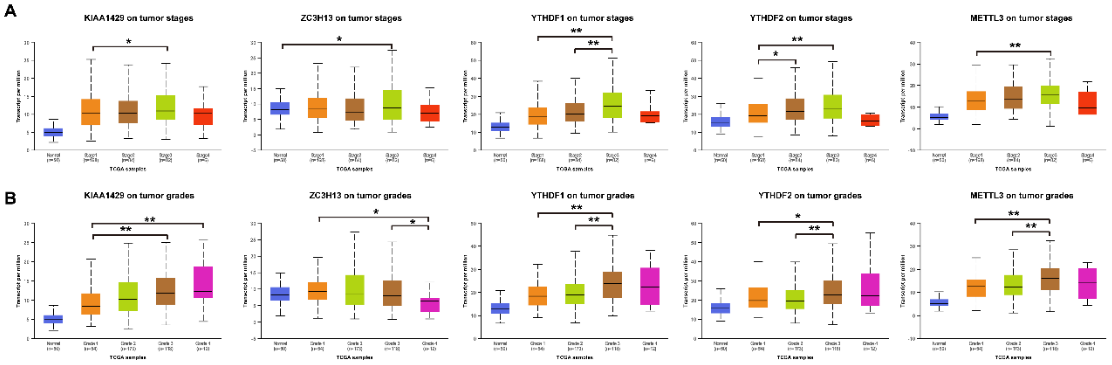

Figure S4. The expression features of the 5 m^{6}A RNA methylation regulators in cases at different stages. (A) The expression features of the 5 m^{6}A RNA methylation regulators in cases at different tumor stages. **(B)** The expression features of the 5 m6A RNA methylation regulators in cases at different tumor grades. *, $P < 0.05$, **, $P < 0.01$.

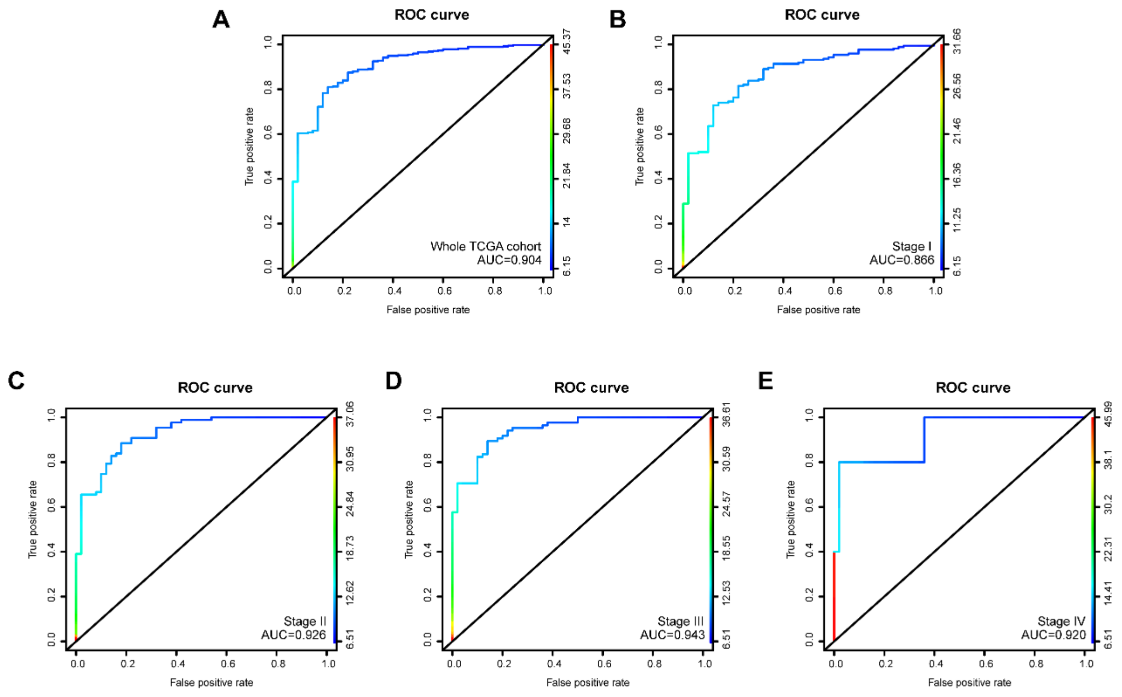

Figure S5. The diagnostic value of YTHDF1 for HCC based on the expression features. (A) The ROC curves of YTHDF1 in the whole TCGA LIHC cohort. **(B-E)** The ROC curves of YTHDF1 in HCC cases at different stages.
